# Occupational Hepatitis B Exposure: A Peek into Indian Dental Students' Knowledge, Opinion, and Preventive Practices

**DOI:** 10.1155/2015/190174

**Published:** 2015-08-30

**Authors:** Sandeep Kumar, Debashish Basak, Amit Kumar, Pralhad Dasar, Prashant Mishra, Arunoday Kumar, Siddharth Kumar Singh, Nitai Debnath, Anjali Gupta

**Affiliations:** ^1^Department of Public Health Dentistry, Sri Aurobindo College of Dentistry, Indore, Madhya Pradesh 453555, India; ^2^Department of Prosthodontics, Hazaribag College of Dental Sciences, Jharkhand 825301, India; ^3^Department of Public Health Dentistry, Sarjug Dental College and Hospital, Bihar 846003, India; ^4^Department of Oral Medicine Diagnosis and Radiology, Sri Aurobindo College of Dentistry, Indore, Madhya Pradesh 453555, India; ^5^Department of Prosthodontics, Dental College, RIMS, Imphal, Manipur 795004, India

## Abstract

*Objective*. To determine the level of knowledge, opinions, and preventive practices followed by dental students against Hepatitis B. The study also explored if any correlation existed between knowledge, opinion, and preventive practices score. *Materials and Methods*. A cross-sectional study was conducted in a dental teaching institution. The subjects comprised 216 dental students. The study was conducted using a pretested, self-administered questionnaire. The questionnaire was prepared to assess knowledge, opinion, and preventive practices against Hepatitis B. Kruskal-Wallis and Kendall Tau test were performed. *Results*. The study found that only 44.4% of the students were vaccinated with Hepatitis B vaccine. 59.3% of the students reported washing their hands after contact with patient's body fluids. 63.9% used personal protective measures like facemask, aprons, head cap, eye shields, and so forth, while treating patients. Median knowledge, opinion, and practice scores were found to be 5.00, 3.00, and 3.00, respectively. Significant correlation was obtained between knowledge and preventive practices score (*r* = 0.385, *p* value <0.0001). *Conclusion*. Effective measures need to be taken to improve preventive practices of the students to prevent them from risk of Hepatitis transmission. Mandatory vaccination against Hepatitis B needs to be implemented.

## 1. Introduction

Hepatitis is an inflammatory disease of the liver and a major global health problem. It is of particular concern in the Asia-Pacific region where chronic HBV infection is prevalent. It is estimated that chronic HBV infection affects more than 350 million people worldwide [1] of whom approximately 75% are Asian [2].

In southeast Asian region, there are estimated 80 million HBV carriers (about 6% of the total population) [3]. India has the intermediate endemicity of Hepatitis B, with Hepatitis B surface antigen prevalence between 2% and 10% among the population studied. The number of carriers in India has been estimated to be over 40 million [4].

Hepatitis B infection can spread through contact with blood, semen, vaginal fluids, and other fluids of someone who already has a Hepatitis B infection. HBV is generally transmitted by unsafe use of therapeutic injections, blood transfusion, shaving by barbers, tattooing, mother-to-child transmission, and unsafe sexual practices [5]. Occupational injuries which expose health care professionals to blood-borne pathogens continue to be an important public health concern. In particular, dentists are at increased risk of exposure to Hepatitis B, Hepatitis C, and HIV [6]. Infections may be transmitted in the dental operatory through several routes including direct contact with blood, oral fluids, or other secretions; indirect contact with contaminated instruments, operatory equipment, or environmental services; or contact with airborne contaminants present in either droplet splatter or aerosols of oral and respiratory fluids [7, 8].

Prevention of spread of Hepatitis B infection is very important in dental practice, as dental students are at a higher risk of transmission of this disease. The dental students should be aware of the risks involved in performing dental procedures and take appropriate steps in preventing the transmission of this disease. Knowledge and awareness about different modes of transmission of this disease are helpful in planning preventive health education programs. This will help to control this disease using preventive strategies. Dental education with special emphasis on infection control protocols will protect dentist as well as patients from unwanted transmission of this disease. With limited literature available to judge the knowledge and opinion of Indian dental students towards preventive strategies to control infectious diseases, this study was conducted with the aim of determining the knowledge, opinion, and preventive practices of the dental students towards the safety measures used in day-to-day practice to prevent spread of Hepatitis B infection in India. The study also explored for any association between the knowledge, opinion, and preventive practice scores.

## 2. Materials and Methods

This was a cross-sectional study carried out in a dental institution in Indore city, Central India. The dental college has an intake of 100 students every year. At the time of data collection, there were 70 students in third year, 85 students in final year, and 80 interns. The data were collected from the final and third year students by the investigator himself in the classroom. The interns filled the questionnaire in their respective departments where they were posted. All the students were invited to take part in the study. The study objectives were explained to the participants and students who were willing to participate in the study were included. An informed consent was sought from the participants prior to the distribution of the questionnaire. All the students who were willing to participate, who were present on the day of study, and who signed the informed consent were included in the study. The final sample comprised of 74 interns (response rate = 92.5%), 79 final year students (response rate = 92.9%), and 63 third year students (response rate = 90.0%). Hence, total sample comprised 216 Indian dental students.

The study population of 216 dental students voluntarily completed a pretested, self-administered, close-ended questionnaire. The questionnaire was prepared in English language and the validity was checked using test-retest method. The reliability was assessed using split-half reliability coefficient test (*p* = 0.82, good reliability). The questionnaire comprised 5 questions to assess knowledge, 5 questions to assess opinion, and 5 questions to assess preventive practices of dental students regarding Hepatitis B transmission and its control. The subjects were asked to tick the most appropriate answer for each question. In addition, the questionnaire also collected information on sociodemographic data. Each correct response was given a score of 1. The “do not know” responses were considered as “no” and were not scored.

Ethical approval was obtained from the Institutional Review Board, Sri Aurobindo Institute of Medical Sciences. All the participants were briefed about the purpose of the study, and all points of the questionnaire were clarified to them. The participants were not permitted to confer with each other. The forms were filled by them in their classroom and completed forms were collected at same time. The students were instructed to undergo vaccination for Hepatitis and follow the preventive practices in order to minimize the risk of transmission.

All the statistical analysis was carried out using the statistical package for social sciences software [SPSS v. 21]. Kolmogorov-Smirnov test was performed to check the normality of the distribution. The data was found to be nonparametric in nature. Kruskal-Wallis test was performed. Correlation between knowledge, opinions, and practices was checked using Kendal Tau correlation analysis. The cutoff level for statistical significance was taken at *p* < 0.05.

## 3. Results

The study population comprised 216 interns, final year students, and third year students. Out of these, 62 were males and 154 were females. A total of 18 males and 45 females from third year, 23 males and 56 females from final year, and 21 males and 53 females from intern batch participated in the study. The mean age of the study population was 21.3 ± 1.7 years.

Nearly 80% of the students had the knowledge that Hepatitis B can be transmitted while performing dental procedures and it can contaminate the body by means of saliva. 80.1% of the students were aware about the incubation period of Hepatitis B and 81.9% were aware that there is a live and active vaccine to prevent Hepatitis B transmission (Table 1).

Only 50% of the students showed a positive opinion in recording Hepatitis B information regarding systemic condition on patients file and believed that health personnel should be trained regarding Hepatitis B. 56% believed that whole medical staff working in contact with blood and body fluids should be vaccinated. 65.7% agreed to the fact that the instruments should be washed right after treatment and before being autoclaved (Table 2).

Only 44.4% of the students were vaccinated with Hepatitis B. 59.3% of the students reported to wash their hands after contact with patient's body fluids. 63.9% used personal protective measures like facemask, aprons, head cap, eye shields, and so forth while treating patients. Only 50.9% check the indicator showing whether or not instruments has been sterilized before using them in any procedure (Table 3).

Knowledge, opinions, and preventive practices scores were calculated separately. Each correct response was given score of 1. The median values for the knowledge, opinion, and preventive practice scores were found to be 5.00, 3.00, and 3.00, respectively. There was a significant difference observed for knowledge score (*p* value < 0.0001) and practice score (*p* value < 0.0001) between the groups (Table 4).

Significant correlation was obtained between knowledge and preventive practices score (*r* = 0.385, *p* value < 0.0001). No significant differences were noted for knowledge-opinion and opinion-preventive practices scores (Table 5).

## 4. Discussion

Hepatitis B is one of the highly infectious diseases whose route of transmission is similar to other infectious diseases. The health care personals should be aware about the epidemiology of this disease in prevention of hepatitis infection. This cross-sectional study was conducted with the objective of determining the knowledge, opinions, and preventive practices of the dental students towards the safety measures used in day-to-day practice to prevent spread of Hepatitis B infection in India. The study also explored if there was any correlation obtained between the knowledge, opinion, and preventive practice scores. A self-administered, pretested questionnaire was used to collect information.

The most effective means to prevent Hepatitis B infection is through vaccination. The protective efficacy of vaccine is well established [9–12]. Recent studies conducted in different parts of the world have shown that there is a substantial reduction in Hepatitis B infections following introduction of vaccination program [13–15]. The most surprising finding of the study was that only 44.4% of the students were vaccinated with Hepatitis B vaccine. Similar results were obtained in study done by Singh et al. [16], in Central India wherein they found that 61.2% of the undergraduate dental students were not vaccinated with Hepatitis B vaccine. In their study, the authors cited that not listing mandatory Hepatitis B vaccination for dental students as an essential requirement might be one of the causes for undergraduate students not undergoing vaccination against Hepatitis B. Despite the recommendations by WHO for vaccinations against Hepatitis B infections, studies conducted on health care workers in other countries have also shown similar findings [17, 18].

The level of knowledge regarding Hepatitis B and its mode of transmission was found to be good. Nearly 80% of the undergraduate students were aware that Hepatitis B can contaminate the body by means of saliva and can be transmitted in routine dental procedures. Similar high level of knowledge regarding Hepatitis B amongst studies done in Indian dental students was reported by Saini et al. [19], Tirounilacandin et al. [20], and Gambhir et al. [21]. Other studies in Pakistan [22] and Oman [23] also reported similar good knowledge of the disease. On the contrary, a lower level of knowledge regarding Hepatitis B and its transmission was reported by studies done by Khalid et al. [24], Koksal et al. [22], and Nicholas et al. [25]. These authors were of the opinion that absence of formal school based education in the country may be the most important reason for lower level of knowledge against this viral disease. However, in our study a higher level of knowledge was obtained which may be attributed to the fact that the study comprised interns, final year students, and third year students who undergo rigorous and repeated revision of this infectious disease through their curriculum.

Our study demonstrated a moderate opinion of the undergraduate dental students towards prevention of Hepatitis B. Only 51.4% of the students showed a positive opinion towards recording Hepatitis information regarding systemic conditions on patients file. 65.7% of the students showed positive behavior towards washing their instruments and autoclaving it prior to use. The authors are of the opinion that development of right opinion is essential for the prevention of transmission of this infectious disease.

In the present study, poor preventive practices were reported by undergraduate dental students. Only 59.3% of the students reported washing their hands after contact with patient's body fluids and only 63.9% used personal protective measures. Only 56.5% of the undergraduate dental students educate their patients about Hepatitis B and offer them to be vaccinated. Similar low preventive practices against Hepatitis B transmission were reported by Afridi et al. [26] and Alavian et al. [27]. The poor preventive services followed by the undergraduate dental students are of significant concern as they are putting themselves as well as their patients to the risk of transmission of Hepatitis B. Dental teaching institutions should focus on strategies to instill positive preventive practices amongst the students and provide opportunities to analyze their own experiences in the dental institution from the perspective of infection control. The approach suggested by Machado-Carvalhais et al. [28] can be adopted as it sensitizes the students to their opinions in order to change their behavior and consequently improve their quality of life.

A significant difference between groups (interns, final year students, and third year students) was noted for knowledge and preventive practices scores with higher knowledge and preventive practices scores found for third year students, followed by final year and then interns. This is suggestive of the fact that third year students have good knowledge and follow better preventive practices against Hepatitis B compared to interns. Similar findings were reported by Singh et al., who found that the overall low mean score obtained for preventive practices by interns is suggestive of the fact that students tend to forget the risk of transmission of infectious diseases such as Hepatitis B over time. Hence, there is a need to instill positive preventive practices amongst the undergraduate students prior to graduation. The topic of infection control requires a proactive approach throughout the course of undergraduate teaching.

Although significant correlations were obtained between knowledge and preventive practices scores, no correlation was found between knowledge-opinion and opinion-preventive practices scores.

The overall knowledge regarding Hepatitis B and its transmission was found to be good among dental students. However, the preventive practices that were followed by them to prevent the risk of transmission of Hepatitis B were not satisfactory. Hence, there is a need for rigorous training of undergraduate students to follow strict infection control guidelines to prevent transmission of Hepatitis B. The finding of this study is an alert for the dental educators to educate the students clearly and comprehensively about Hepatitis B and its transmission in dental practice.

Immunization of dental health care professionals is one of the most effective means for prevention and transmission of Hepatitis B. Immunization substantially reduce both the number of dental health care persons susceptible to these diseases and the potential for disease transmission to other dental health care personnel and patients [29]. Hence, there should be an immunization schedule for students and dental health care workers which should be listed by educational institutions and mandatorily followed.

The study was carried out in a single institution and, hence, generalizability of the results can be questioned. A major limitation of this study includes cross-sectional nature of the study design. We found that the third year students had better knowledge and followed better preventive practices and believed that as the years of academic study increase, the students tend to forget things and ignore the protocols, but due to the cross-sectional nature of the study design we cannot generalize the findings and say that always similar results will be obtained. There could be other reasons also which the authors could not explain and this suggests that further longitudinal studies involving participants from different institutions are needed before the results can be generalized.

## 5. Conclusions

The overall knowledge score regarding Hepatitis B virus was found to be good. The overall knowledge and preventive practices followed were significantly better for third year students. A weak correlation was obtained between knowledge and preventive practices score. Majority of the children were not vaccinated against Hepatitis B which warrants the need for drafting effective policies to protect the undergraduate students from risk of Hepatitis B.

## Figures and Tables

**Table 1:** Knowledge of the students regarding Hepatitis B.

Sr. number	Questions	Response	*N* (%)
1	Can Hepatitis B be transmitted while performing dental procedures?	Yes	178 (82.4%)
		No	26 (12%)
		Do not know	12 (5.6%)

2	Can Hepatitis B contaminate the body by means of saliva?	Yes	176 (81.5%)
		No	32 (14.8%)
		Do not know	8 (3.7%)

3	Is Hepatitis B a RNA virus?	Yes	187 (86.6%)
		No	18 (8.3%)
		Do not know	11 (5.1%)

4	Is the incubation period of Hepatitis B about 50–80 days?	Yes	173 (80.1%)
		No	30 (13.9%)
		Do not know	13 (6.0%)

5	Is Hepatitis B active and live vaccine?	Yes	177 (81.9%)
		No	22 (10.2%)
		Do not know	17 (7.9%)

**Table 2:** Opinions of students regarding Hepatitis B.

Sr. number	Questions	Response	*N* (%)
1	Do you think Hepatitis information regarding systemic condition must be recorded on patients file?	Yes	111 (51.4%)
		No	65 (30.1%)
		Do not know	40 (18.5%)

2	Do you think whole medical staff working with blood and body fluids in contact should be vaccinated?	Yes	121 (56%)
		No	66 (30.6%)
		Do not know	29 (13.4%)

3	Do you think the instruments should be washed right after treatment and before autoclaving?	Yes	142 (65.7%)
		No	42 (19.4%)
		Do not know	32 (14.8%)

4	Do you think health personnel should be trained regarding Hepatitis B?	Yes	131 (60.6%)
		No	58 (26.9%)
		Do not know	27 (12.5%)

5	Do you think, after instrument injuries, nonvaccinated health care workers should have immunoglobulin installation and three-dose vaccination programme?	Yes	104 (48.1%)
		No	75 (34.7%)
		Do not know	37 (17.1%)

**Table 3:** Preventive practices of students regarding Hepatitis B.

Sr. number	Questions	Response	*N* (%)
1	Have you been vaccinated with all doses of Hepatitis B?	Yes	96 (44.4%)
		No	88 (40.7%)
		Do not know	32 (14.8%)

2	Do you inform the patients about Hepatitis and offer them to be vaccinated?	Yes	122 (56.5%)
		No	80 (37.0%)
		Do not know	14 (6.5%)

3	Do you use personal protective measures like facemask, aprons, head cap, eye shields, and so forth while treating patients?	Yes	138 (63.9%)
		No	57 (26.4%)
		Do not know	21 (9.7%)

4	Do you wash your hands after contact with patients body fluids?	Yes	128 (59.3%)
		No	67 (31.0%)
		Do not know	21 (9.7%)

5	Do you check the indicator showing whether or not instruments have been sterilized before using them in any procedure?	Yes	110 (50.9%)
		No	72 (33.3%)
		Do not know	34 (15.7%)

**Table 4:** Median (minimum–maximum) of knowledge, opinions, and preventive practices scores regarding infection control.

Group	Knowledge Median (min–max)	Opinion Median (min–max)	Preventive practices Median (min–max)
Interns	4.00 (1.00–5.00)	3.00 (1.00–5.00)	2.00 (1.00–5.00)
4th year	5.00 (1.00–5.00)	3.00 (1.00–5.00)	3.00 (1.00–5.00)
3rd year	5.00 (1.00–5.00)	2.00 (1.00–5.00)	4.00 (1.00–5.00)
Total	5.00 (1.00–5.00)	3.00 (1.00–5.00)	3.00 (1.00–5.00)
*p* value^#^	<0.0001^*∗∗∗*^	0.106	<0.0001^*∗∗∗*^

^*∗∗∗*^Highly significant difference.

^#^Kruskal-Wallis test performed.

**Table 5:** Correlation among knowledge, opinion, and preventive practices scores.

Variables	Correlation coefficient	*p* value
Knowledge-opinion	−0.040	0.491
Opinion-preventive practices	−0.052	0.369
Knowledge-preventive practices	0.385	<0.0001^*∗∗∗*^

^*∗∗∗*^Correlation is significant at 0.01 level (2-tailed).
